# Exploring the Phase-Locking Mechanisms Yielding Delayed and Anticipated Synchronization in Neuronal Circuits

**DOI:** 10.3389/fnsys.2019.00041

**Published:** 2019-08-21

**Authors:** Leonardo Dalla Porta, Fernanda S. Matias, Alfredo J. dos Santos, Ana Alonso, Pedro V. Carelli, Mauro Copelli, Claudio R. Mirasso

**Affiliations:** ^1^System Neuroscience Group, Institut d'Investigacions Biomèdiques August Pi i Sunyer, Barcelona, Spain; ^2^Instituto de Física, Universidade Federal de Alagoas, Maceió, Brazil; ^3^Departamento de Física, Universidade Federal de Pernambuco, Recife, Brazil; ^4^Instituto de Física Interdisciplinar y Sistemas Complejos (IFISC, UIB-CSIC), Palma, Spain

**Keywords:** neuronal oscillations, neuronal dynamics, neuronal motifs, synchronization, anticipated synchronization

## Abstract

Synchronization is one of the brain mechanisms allowing the coordination of neuronal activity required in many cognitive tasks. Anticipated Synchronization (AS) is a specific type of out-of-phase synchronization that occurs when two systems are unidirectionally coupled and, consequently, the information is transmitted from the sender to the receiver, but the receiver leads the sender in time. It has been shown that the primate cortex could operate in a regime of AS as part of normal neurocognitive function. However it is still unclear what is the mechanism that gives rise to anticipated synchronization in neuronal motifs. Here, we investigate the synchronization properties of cortical motifs on multiple scales and show that the internal dynamics of the receiver, which is related to its free running frequency in the uncoupled situation, is the main ingredient for AS to occur. For biologically plausible parameters, including excitation/inhibition balance, we found that the phase difference between the sender and the receiver decreases when the free running frequency of the receiver increases. As a consequence, the system switches from the usual delayed synchronization (DS) regime to an AS regime. We show that at three different scales, neuronal microcircuits, spiking neuronal populations and neural mass models, both the inhibitory loop and the external current acting on the receiver mediate the DS-AS transition for the sender-receiver configuration by changing the free running frequency of the receiver. Therefore, we propose that a faster internal dynamics of the receiver system is the main mechanism underlying anticipated synchronization in brain circuits.

## 1. Introduction

Brain rhythms have been extensively studied and related to plenty of neurocognitive tasks in the last decades (Buzsáki, 2006). According to the communication through coherence hypothesis (Fries, 2005), neuronal oscillation locked at the appropriate phase may facilitate information transmission between brain regions. Despite the fact that the phase relations are associated to synaptic delays between distant regions, non-linear ingredients as inhibition and external noise acting locally can also control the phase relation between coupled areas. When the oscillations of certain area A influence and lock those of another area B, it is expected that the phase between A and B (defined as ϕ_*B*_ − ϕ_*A*_) be positive [a regime we refer to as delayed synchronization (DS)]. However, a counterintuitive phase relation was observed between cortical regions in primates (Brovelli et al., 2004; Salazar et al., 2012). Under certain circumstances, a directional influence between two cortical areas is accompanied by a negative time delay (i.e., by a negative phase difference). This phenomenon has been explained by the concept of anticipated synchronization (AS) (Voss, 2000; Matias et al., 2014).

As proposed by Voss, two identical autonomous dynamical systems unidirectionally coupled in a sender-receiver configuration can exhibit anticipated synchronization if the receiver is subject to a delayed negative self-feedback:

(1) $$\begin{array}{lll} \overset{∙}{\text{S}} & = & \text{f}\left(\text{S}\left(t\right)\right),\\ \overset{∙}{\text{R}} & = & \text{f}\left(\text{R}\left(t\right)\right)+K\left[\text{S}\left(t\right)-\text{R}\left(t-t_{d}\right)\right], \end{array}$$

where **f**(*S*) is a vector function that describes the autonomous dynamical system, *K* is the coupling matrix and the delayed term **R**(*t* − *t*_*d*_) is the self-feedback (Voss, 2000). The solution **R**(*t*) = **S**(*t* + *t*_*d*_) characterizes the regime of anticipated synchronization and has been verified in a variety of theoretical (Voss, 2000, 2001a,b, 2016, 2018; Masoller and Zanette, 2001; Hernández-García et al., 2002; Ciszak et al., 2003; Kostur et al., 2005; Sausedo-Solorio and Pisarchik, 2014) and experimental (Sivaprakasam et al., 2001; Tang and Liu, 2003; Ciszak et al., 2009; Stepp and Turvey, 2017) studies.

The AS regime has been reported in systems without the explicit delay term. For example, for a specific parameter mismatch between the sender and the receiver system that gives a first-order approximation to the delayed coupling (Corron et al., 2005). AS has also been reported in a chain consisting of a sender and two receivers with switching parameters (Pyragienè and Pyragas, 2015), between two Hodgkin-Huxley neurons with different depolarization parameters (Simonov et al., 2014) and in the presence of an inhibitory loop mediated by an interneuron with a free-running frequency greater than the others (Matias et al., 2017). It has also been shown that AS may appear between two neuron models directly coupled provided that the mean frequency of the free receiver is greater than the mean frequency of the sender with (Hayashi et al., 2016) and without the explicit time-delay (Pyragienè and Pyragas, 2013; Dima et al., 2018). AS has been verified in a system in which the delayed feedback has been replaced by a simple, low-order all-pass filter (Pyragiene and Pyragas, 2017). More recently, a novel theoretical viewpoint based on the mathematical object called canard, has been used to explain anticipation in excitable systems (Ersös et al., 2019)

In neuronal rhythms the relative phase between two coupled regions is an important characteristic of the dynamics since it can modulate the information flow of an unexpected stimuli (Barardi et al., 2014). Here we investigate the mechanisms underlying the transition from positive to negative phase locking (or equivalently the transition between the DS and AS regimes) on multiple scales. For synchronized systems, it is equivalent to define the phase relation or the time delay between peaks of activity. We simulate three different motifs of unidirectionally coupled systems in which the negative delayed feedback of Equation (1) is replaced by a synaptic inhibitory loop. We extend previous results for three coupled neurons (Matias et al., 2011) and cortical-like populations (Matias et al., 2014), showing that the AS-DS transition can be mediated not only by the inhibitory synaptic conductance but also by the external stimulus at the receiver. We also show that a neural mass model, known to exhibit zero-lag synchronization (Gollo et al., 2014), can operate in the anticipated synchronization regime and the AS-DS transition can be mediated by the stimuli acting on the receiver as well by an inhibitory loop. Moreover, we show that when the sender and receiver are uncoupled the inhibitory loop and an external current acting at the receiver system change its internal dynamics which is reflected in its free running frequency. More important, we found that the phase difference between the sender and the receiver decreases when the free running frequency of the receiver increases. Therefore, we propose that for an excitation/inhibition balance and biologically plausible parameters a faster internal dynamics of the receiver as compared to the emitter is the mechanism underlying AS. We also suggest that the DS-AS transition studied here could be mediated by any parameter that turns the internal dynamics of the free-receiver faster (or equivalently the free-running frequency of the sender slower) and could also account for delay compensation in cortical systems.

## 2. Materials and Methods

### 2.1. Microcircuit

The model for the 3-neurons motif is the one proposed by Matias et al. (2011). Neurons are described by the Hodgkin-Huxley model (Hodgkin and Huxley, 1952) composed by the currents *I*_*Na*_, *I*_*K*_ and *I*_*L*_:

(2) $$\begin{array}{lll} C_{m}\frac{dV}{dt} & = & I_{Na}+I_{K}+I_{L}+I_{ext}+I_{syn}, \end{array}$$

where *C*_*m*_ = 9πμF is the membrane capacitance of a 30 × 30 × π μm^2^ equipotential patch of membrane, *I*_*ext*_ is the external constant current that sets the neuron excitability and *I*_*syn*_ is the pre-synaptic current.

The ion channels follows the Hodgkin-Huxley formalism: *dx*/*dt* = ϕ[α_*x*_(*V*)(1 − *x*) − β_*x*_(*V*)*x*], being ϕ = 1 the temperature factor. The sodium current $I_{Na}=g_{Na}m^{3}h\left(V_{Na}-V\right)$ has a maximal conductance *g*_*Na*_ = 1080π mS, and rate constants $\alpha_{m}\left(V\right)=0.1\left(25-V\right)/\left(e^{0.1\left(25-V\right)}-1\right)$, $\beta_{m}\left(V\right)=4e^{-V/18}$, $\alpha_{h}\left(V\right)=0.07e^{-V/20}$ and $\beta_{h}\left(V\right)=1/\left(e^{0.1\left(30-V\right)}+1\right)$. The delayed-rectifier potassium current $I_{K}=g_{k}n^{4}\left(V_{K}-V\right)$ has a maximal conductance *g*_*K*_ = 324π mS and rate constants $\alpha_{n}\left(V\right)=0.01\left(10-V\right)/\left(e^{0.1\left(10-V\right)}-1\right)$ and $\beta_{n}\left(V\right)=0.125e^{-V/80}$. The leakage current *I*_*L*_ = *g*_*L*_(*V*_*L*_ − *V*) has a maximal conductance *g*_*L*_ = 2.7π mS. The reversal potentials are *V*_*Na*_ = 115 mV, *V*_*K*_ = −12 mV and *V*_*L*_ = 10.6 mV. In all the expressions above, *V* is measured in mV.

**Figure 1:**
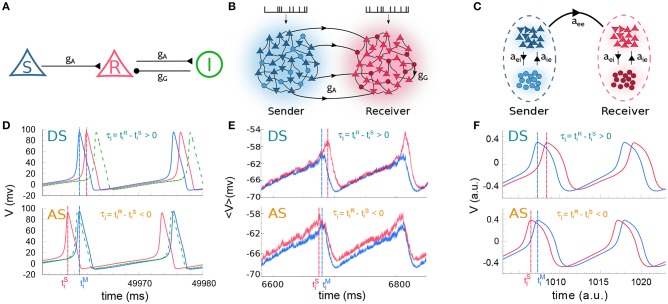
Cortical motif circuits. Schematic representation of the models: **(A)** 3-neuron motif, **(B)** two cortical populations, and **(C)** two neural masses. **(D)** Membrane potential of three neurons in a regime of delayed-synchronization (DS, top) and anticipated-synchronization (AS, bottom). **(E,F)** Average membrane potential in a regime of DS (top) and AS (bottom), for populations and neural masses, respectively. For the sake of clarity, the data in **(D–F)** correspond to simulations where the external parameter was varied. The parameters used were: **(D)** *g*_*G*_ = 20.0 nS with ${\text{I}}_{ext}^{R}=280$ pA (DS) and ${\text{I}}_{ext}^{R}=320$ pA (AS); **(E)** *g*_*G*_ = 8.0 nS with $\lambda_{Ext}^{R}=2.8$ kHz (DS) and $\lambda_{Ext}^{R}=3.0$ kHz (AS); **(F)** *g*_*G*_ = 1.4 with ${\text{I}}_{ext}^{R}=0.1$ (DS) and ${\text{I}}_{ext}^{R}=0.75$ (AS). For **(A–C)** excitatory and inhibitory neurons are represented by triangle and circle, respectively. Also, the colors used in **(D–F)**, respectively, stand for the same colors as in **(A–C)**.

The synaptic current *I*_*syn*_ = *g*_*syn*_*r*(*V*_*syn*_ − *V*) comprises the gating variable *r* following *dr*/*dt* = α[*T*](1 − *r*) − β*r*, where α and β are rate constants and $\left[T\right]\left(V_{pre}\right)=T_{max}/\left(1+e^{\left(V_{p}-V_{pre}\right)/K_{p}}\right)$ is the neurotransmitter concentration in the synaptic cleft. In this model AMPA (A) and GABA_A_ (G) are the excitatory and inhibitory synapses, respectively. The parameters concerning the synapses are: α_*A*_ = 1.1 (mM^−1^ms^−1^), β_*A*_ = 0.19 (ms^−1^), α_*G*_ = 5.0 (mM^−1^ms^−1^), β_*G*_ = 0.30 (ms^−1^), *T*_*max*_ = 1 mM^−1^, *K*_*p*_ = 5 mV, *V*_*p*_ = 62 mV, *V*_syn,AMPA_ = 60 mV and *V*_syn,GABA_ = −20 mV. The three neurons are: sender, receiver, and interneuron. The sender projects excitatory synapses onto the receiver. The receiver projects excitatory and receives inhibitory synapses from the interneuron (see Figure 1). We kept *g*_*A*_ = 10.0 nS, $I_{ext}^{S}=280.0$ pA and $I_{ext}^{Inter}=280.0$ pA fixed throughout all the simulations. The free parameters of this model are *g*_*G*_ and $I_{ext}^{R}$. When $g_{G}^{R}$ was varied, $I_{ext}^{R}=280$ pA was kept fixed, whereas when $I_{ext}^{R}$ was varied, $g_{G}^{R}=20.0$ nS was kept fixed.

### 2.2. Neuronal Populations

For two cortical populations we follow the ideas proposed by Matias et al. (2014). Each population, sender (S) and receiver (R), is composed of excitatory (80%) and inhibitory (20%) neurons, whose dynamics is described by the Izhikevich model (Izhikevich, 2003):

(3) $$\begin{array}{lll} \frac{dv}{dt} & = & 0.04v^{2}+5v+140-u+\sum I_{syn}, \end{array}$$

(4) $$\begin{array}{lll} \frac{du}{dt} & = & a\left(bv-u\right), \end{array}$$

where *v* and *u* stands for the membrane potential of the neuron and the membrane recovery variable (activation of K^+^ and inactivation of Na^+^ ionic currents), respectively. *a, b, c* and *d* are dimensionless parameters that account for the firing patterns heterogeneity which are randomly distributed accordingly to the neuron's nature. For excitatory neurons *a* = 0.02, *b* = 0.20, *c* = −65 + 15σ^2^ and *d* = 8 − 6σ^2^, whereas for inhibitory neurons *a* = 0.02 + 0.08σ, *b* = 0.25 − 0.05σ, *c* = −65.0 and *d* = 2.0. σ ∈ (0, 1) is a random variable. If a spike occurs, i.e., *v* ⩾ −30mV, *v* is reset to *c* and *u* to *u* + *d*.

The synaptic transmissions are mediated by excitatory AMPA (A) and inhibitory GABA_*A*_ (G). The pre-synaptic current is described as *I*_*syn*_ = −*g*_*syn*_*r*(*v* − *V*_*syn*_), where *V*_*A*_ = 0 mV and *V*_*G*_ = −65 mV. *g*_*syn*_ is the maximal conductance, *g*_*A*_ for excitatory and *g*_*G*_ for inhibitory synapses. *r* is the gating variable and follows a first-order kinetic dynamics: $\tau_{syn}dr/dt=-r+D\underset{j}{\sum }\delta \left(t-t_{j}\right)$, where τ_*A*_ = 5.26 ms, τ_*G*_ = 5.60 ms and the summation over *j* stands for the neighbor's pre-synaptic spikes at the previous time steps {*t*_*j*_}. D is taken, without loss of generality, equal to 0.05.

The populations S and R are composed of 500 neurons each, among which 80% are pyramidal cells and 20% inhibitory interneurons. In the S population, each neuron receives 50 randomly chosen synapses from other neurons with excitatory conductances $g_{A}^{S}=0.5$ nS and inhibitory conductances $g_{G}^{S}=4$ nS, which remained fixed throughout the simulations. In the R population, each neuron receives 10 inhibitory synapses ($g_{G}^{R,I}=4$ nS for inhibitory neurons and $g_{G}^{R}$ for excitatory neurons) and 40 excitatory synapses ($g_{A}^{R}=0.5$ nS). For both populations, no autapses are allowed. The connectivity between S and R populations is such that all neurons within the R population receive 20 randomly chosen excitatory synapses from the S population ($g_{A}^{SR}=0.5$ nS, unless otherwise specified). Also, all neurons, within the S and R populations, are subject to an independent noisy spike train described by a Poisson distribution with rate λ. The input mimics excitatory synapses from neurons that are not included in the populations, with a maximal conductance *g*_*A*_ = 0.5 nS. Without loss of generality, we assume for the S population λ^*S*^ = 2.4 kHz. So, the free parameters for this model are $g_{G}^{R}$ and $\lambda_{ext}^{R}$. When $g_{G}^{R}$ was varied, $\lambda_{ext}^{R}=2.4$ kHz was kept fixed, whereas when $\lambda_{ext}^{R}$ was varied, $g_{G}^{R}=8.0$ nS was kept fixed.

### 2.3. Neural Mass Models

The large-scale circuit model is the one used in Gollo et al. (2014). Briefly, the neural mass model (NMM) is composed by three state variables: *V* is the mean membrane potential of pyramidal neurons; *Z*, the mean membrane potential for interneurons; and *W* is the average number of open potassium ion channels. Here we made use of two ensembles *i* = *S, R*, namely Sender and Receiver. The equations for the dynamics are given by:

(5) $$\begin{array}{l} \frac{dV^{i}(t)}{dt}=-\{g_{Ca}+(1.0-C_{ji})r_{NMDA}a_{ee}Q_{V}^{i}(t)\\ \;+C_{ji}r_{NMDA}a_{ee}<Q_{V}^{j}(t-\tau )>\}m_{Ca}(V^{i}(t)-V_{Ca})\\ \;-g_{K}W^{i}(t)(V^{i}(t)-V_{K})-g_{L}(V^{i}(t)-V_{L})\\ \;-\{g_{Na}m_{Na}+a_{ee}(1.-C_{ji})Q_{V}^{i}(t)\\ \;+C_{ji}a_{ee}<Q_{V}^{j}(t-\tau )>\}(V^{i}(t)-V_{Na})\\ \;-a_{ie}Z^{i}(t)Q_{Z}^{i}+a_{ne}I_{ext}^{E}, \end{array}$$

(6) $$\begin{array}{lll} \frac{dZ^{i}\left(t\right)}{dt} & = & b\left(a_{ni}I_{ext}^{I}+a_{ei}V^{i}\left(t\right)Q_{V}^{i}\left(t\right)\right), \end{array}$$

(7) $$\begin{array}{lll} \frac{dW^{i}\left(t\right)}{dt} & = & \frac{\phi \left\{m_{K}-W^{i}\left(t\right)\right\}}{\tau_{W}}, \end{array}$$

(8) $$\begin{array}{l} m_{ion}=0.5\left[1+\tanh\left(\frac{V^{i}(t)-T_{ion}}{\delta_{ion}}\right)\right], \end{array}$$

(9) $$\begin{array}{l} Q_{V}^{i}(t)=0.5Q_{Vmax}\left[1+\tanh\left(\frac{V^{i}(t)-V_{T}}{\delta_{V}}\right)\right], \end{array}$$

(10) $$\begin{array}{l} Q_{Z}^{i}(t)\;=0.5Q_{Zmax}\left[1+\tanh\left(\frac{Z^{i}(t)-Z_{T}}{\delta_{Z}}\right)\right], \end{array}$$

where *m*_*ion*_ and $Q_{V,Z}^{i}$ are the fraction of open channels and neuronal firing rates, respectively.

The parameters are: *g*_*Ca*_ = 1.1, *g*_*K*_ = 2, *g*_*L*_ = 0.5, *g*_*Na*_ = 6.7, *r*_*NMDA*_ = 0.25, ϕ = 0.7, τ_*W*_ = 1.0, *b* = 0.1, *T*_*K*_ = 0, *T*_*Ca*_ = −0.01, *T*_*Na*_ = 0.3, δ_*K*_ = 0.3, δ_*Na*_ = 0.15, δ_*Ca*_ = 0.15, *V*_*Ca*_ = 1, *V*_*K*_ = −0.7, *V*_*L*_ = −0.5, *V*_*Na*_ = 0.53, *V*_*T*_ = *Z*_*T*_ = 0, *Q*_*Vmax*_ = *Z*_*Vmax*_ = 0, δ_*V*_ = δ_*Z*_ = 0.65, *a*_*ei*_ = 2, *a*_*ee*_ = 0.4, *a*_*ne*_ = 1, *a*_*ni*_ = 0.4 and $I_{ext}^{I}=0.02$ (here the upper index stands for the inhibitory sub-population in both sender and receiver groups). All quantities are dimensionless. In the sender, the maximal conductance from interneurons to pyramidal neurons as well as the external current in the excitatory neurons are kept constant, $a_{ie}^{S}=2.4$ and $I_{ext}^{S}=0.20$, respectively. Thus, $a_{ie}^{R}$ and $I_{ext}^{R}$ are the free parameters of this model. The coupling (*C*_*ji*_) between the two neural masses are: *C*_*RS*_ = 0 and *C*_*SR*_ = 0.4, thus guaranteeing an unidirectional *master-slave* configuration between S and R. When $a_{ie}^{R}$ was varied $I_{ext}^{R}=0.3$ was kept fixed, whereas when $I_{ext}^{R}$ was varied $a_{ie}^{R}=1.4$ was kept fixed.

### 2.4. Numerical Methods and Data Analysis

The model for 3-neurons-motif was implemented in a C code and simulated using a forth-order Runge-Kutta method with a time step of 5 × 10^−3^ ms. The equations for the neuronal populations and neural masses were implemented in a C++ code and simulated using the Euler method, with a time step of 5 × 10^−2^ ms for neuronal populations and of 10^−3^ (arbitrary units) for neural masses. To compute the mean response of the membrane potential in NMMs we averaged over 10 realizations of the initial conditions while for the populations we averaged over 10 realizations of the external noise, network connectivity and neuron parameters.

The population membrane potential was estimated from the average value of the individual cell's membrane potential (a variable comparable to the local field potential (LFP) recorded in experiments). In order to smooth the noisy signal of the membrane potential we used a Butterworth low-pass filter of fourth order and cutoff frequency of 5 rad/s. From the filtered signal we and extract the peak at times *t*_*i*_. We then calculate the time delay in each cycle as $\tau_{i}=t_{i}^{R}-t_{i}^{S}$ (see Figure 1E). Also, the main frequency (ω) of the neuronal population was obtained detecting the peak of the power spectrum computed via the Fast-Fourier Transform (FFT). For the 3-neurons-motif and also for NMMs, we compute the period in each cycle as the difference between consecutive peaks at times *t*_*i*_, i.e., $T_{i}^{*}=t_{i+1}^{*}-t_{i}^{*}$ (^*^ = R,S). The time delay is estimated as in the case of neuronal populations.

## 3. Results

We investigated the phase locking characteristics, or equivalently in this case the synchronization characteristics, of a unidirectionally coupled system A → B. Our main analysis assumes that the two dynamical nodes A and B are phase locked such that computing the phase difference is equivalent to compute the time difference between peaks. For this reason, from now on we talk about synchronization instead of phase locking. In a delayed synchronization condition, the time difference between the spikes in A and B is positive (A leads B), i.e., the pulse in B occurs after the pulse in A. In the less intuitive case of anticipated synchronization, the pulse in B precedes that in A, yielding a negative time difference.

### 3.1. The DS-AS Transition Can be Mediated by the External Input at the Receiver

In this section we analyze how an input in the receiver side can induce a transition from DS to AS (or vice versa) for the three systems under study.

#### 3.1.1. 3-Neuron Motif

We start by studying the spiking dynamics of a circuit composed of 3 Hodgkin-Huxley cells coupled by chemical synaptic connections as in Matias et al. (2011) (for a schematic representation of the network architecture see Figure 1A and the Methods section for more details). The sender (S) neuron excites the receiver (R) neuron which also participates in an inhibitory loop mediated by an interneuron. For the simplest situation in which *g*_*G*_ = 20 nS and all cells receive the same external current $I_{ext}^{S}=I_{ext}^{R}=I_{ext}^{Inter}=280$ pA, the neurons synchronize in the expected DS regime which exhibits the expected pre-post spike order. The neuronal time series show that the R neuron fires right after the S neuron (top panels of Figure 1D).

For an external constant current, after a transient time and within a synchronized state, the spike time difference converges to a constant value τ = τ_*i*_ that is independent of the initial conditions. By definition, DS is characterized by a positive τ (or phase difference) and AS by a negative τ (or phase difference). As we increase the inhibitory conductance *g*_*G*_, the spike time difference τ decreases, eventually changing sign and reaching negative values (see Matias et al., 2011). When this happens, the S and R neurons fire in a post-pre order (see bottom panel of Figure 1D for *g*_*G*_ = 20 nS and $I_{ext}^{R}=320$ pA) characteristic of the AS regime. A similar effect can be obtained by increasing the external current at the receiver $I_{ext}^{R}$, without changing the value of the conductance *g*_*G*_, as discussed below.

**Figure 2:**
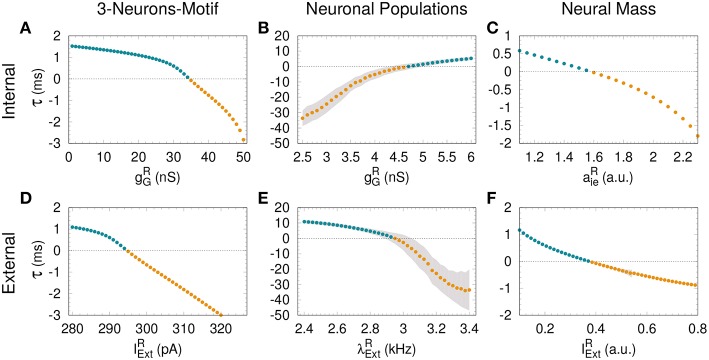
Assessing anticipated and delayed synchronization in cortical motifs. For all the models—3-neurons-motif (left), neuronal populations (middle) and neural masses (right)—a transition from delayed synchronization (DS, τ > 0, cyan dots) to anticipated synchronization (AS, τ < 0, yellow dots) is possible increasing the inhibition in the receiver **(A–C)**; or increasing the external stimulus **(D–F)**. Gray shadow represents the standard deviation over 10 runs (see Methods). For the parameters used here see Methods.

The dependence of τ with *g*_*G*_ has been previously studied in Matias et al. (2011) for a large region of parameter space. The transition from DS to AS is continuous and smooth, and τ is a function of *g*_*G*_ (see Figure 2A). Here we extend these findings, showing that a similar transition from DS to AS can be mediated by a different mechanism, namely increasing the external current of the R neuron. Starting from a DS regime in which $I_{ext}^{S}=I_{ext}^{R}$ and *g*_*G*_ = 20 nS, the spiking time difference τ decreases as we increase $I_{ext}^{R}$ (see Figure 2D).

#### 3.1.2. Neuronal Populations

Similar patterns of out-of-phase synchronization have been reported for two unidirectionally coupled cortical-like populations composed of hundreds of neurons connected by chemical synapses (see Matias et al., 2014, Figure 1B and Methods for more details). Each population is composed by excitatory and inhibitory neurons, each of them receiving an independent Poisson input with rate λ, which accounts for excitatory synapses from neurons that are not included in the population. By construction, both Sender (S) and receiver (R) populations have inhibitory loops within the populations. Depending on the synaptic conductances and the external Poisson current, the mean activity of all neurons in each population may exhibit an oscillatory component. Moreover, the activity of the S and R populations can synchronize with a specific phase difference or equivalently time difference. As an example, it can be seen in the top panel of Figure 1E that if the neurons from both populations receive a noisy spike train with distribution rate $\lambda_{ext}^{S}=2.4$ kHz and $\lambda_{ext}^{R}=2.8$ kHz and the inhibitory synaptic conductance are $g_{G}^{S}=4.0$ nS and $g_{G}^{R}=8.0$ nS, the system operates in a DS regime. The peak of the mean activity 〈*V*〉 of the S population occurs before the peak of the R population. For the populations the spike time difference in each cycle *i* defined as $\tau_{i}=t_{i}^{R}-t_{i}^{S}$, where $t_{i}^{S}$ is the peak of the mean activity of all neurons in S at the *i*−*th* cycle. Due to the noise, we can also define a spike time difference τ as the mean of τ_*i*_ averaged over many cycles. If we increase the external Poisson input at the R population, τ decreases and the system reaches an AS regime (see the bottom panel of Figure 1E for $\lambda_{ext}^{R}=3.0$ kHz). The transition from DS to AS is continuous and smooth (see Figure 2E). To our knowledge, this is the first time that the DS-AS transition mediated by the level of noise in the receiver system is reported. Conversely, the dependence of τ with the inhibitory conductance has been previously reported in Matias et al. (2014) (see Figure 2B).

#### 3.1.3. Neural Mass Models

To further investigate the robustness of the relationship between the local parameters of the receiver system and the existence of anticipated synchronization we also studied the case of two unidirectionally coupled neural mass models, which represents a reduced model of spontaneous cortical dynamics. The neural mass model used here accounts for the neuronal population dynamics and uses three non-linear differential equations per node: one equation for the excitatory subpopulation, one for the inhibitory subpopulation and one for the number of open potassium channels. By its own definition the model has an inhibitory loop mediated by an effective conductance *a*_*ei*_ from excitatory to inhibitory neurons and *a*_*ie*_ from inhibitory to excitatory neurons (see Figure 1C). We find, as in previous cases, that two neural masses unidirectionally connected may synchronize and the inhibitory conductance *a*_*ie*_ as well as the external current $I_{ext}^{R}$ at the receiver node can control the phase-locking difference between them (see Figure 1F). The transition from DS to AS via zero-lag can be obtained by increasing *a*_*ie*_ or $I_{ext}^{R}$ (see Figures 2C,F).

### 3.2. The Frequency of the Free-Running Receiver Serves as a Mechanism Underlying the Synchronization Transition

Based on previous work on anticipated synchronization in the framework of Equation (1) (Pyragienè and Pyragas, 2013; Hayashi et al., 2016; Dima et al., 2018), we studied the effect that an inhibitory connection and the external current plays in determining the frequency of the receiver system when the sender and the receiver are uncoupled (the receiver free-running frequency ω_*R*_). In fact, we find that both the inhibitory conductance and the external stimuli modify the receiver internal dynamics. More important, we find a correlation between the transition from DS to AS regime and the increase of the receiver free running frequency ω_*R*_, and consequently in ω_*R*_ − ω_*S*_ since ω_*S*_ is fixed. We find that the DS-AS transition can be mediated by a change in the receiver free running frequency in multiple scales and by two different parameters (see **Figure 4**). Therefore, we propose that a faster free-running frequency of the receiver is the mechanism yielding anticipation. Similarly, it is also possible to obtain AS by keeping the Receiver intact and slowing-down the Sender free-running frequency.

**Figure 3:**
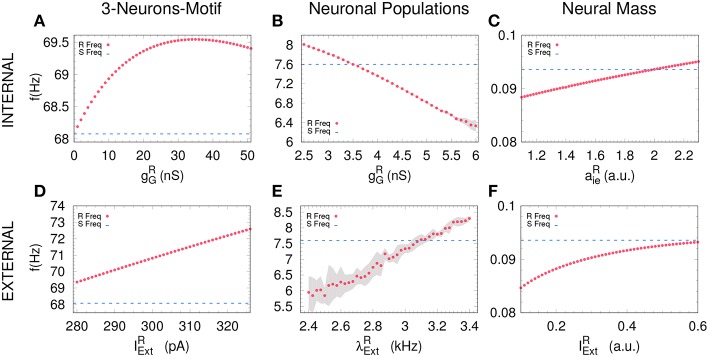
Receiver free-running frequency. Assessing the free-running frequency of the receiver when uncoupled from the sender for all the models. **(A–C)** show how the receiver's frequency changes when varying the internal parameter while **(D–F)** when changing an external parameter. Blue dashed line represents the sender's natural frequency and magenta dots represents the receiver's frequency. Gray shadow represents the standard deviation over 10 runs (see Methods). For the parameters used here see Methods.

**Figure 4:**
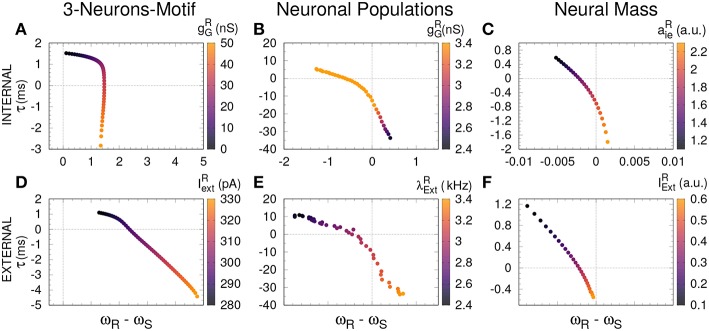
Delayed and anticipated synchronization as a function of the free-running frequency. For all the cases, τ (y-axis) was computed in a coupled system, while the frequency difference (x-axis) was computed for an uncoupled system; the parameters used in both cases are specified by the color code. **(A–C)** Internal parameter vs. frequency differences and **(D–F)** external parameter vs. frequency differences, for 3-neurons-motif, neuronal populations, and neural masses, respectively. Horizontal and vertical dashed lines represent zero−lag synchronization (coupled system) and a perfect match between sender and receiver free-running frequencies, respectively. For the parameters used here see Methods.

An increasing external stimuli increases the frequency of the uncoupled receiver for the three systems (see Figures 3D–F). On the contrary, an increasing inhibitory conductance increases the receiver frequency for the 3-neuron motif and the neural mass model but decreases the free-running frequency in the case of neuronal populations (compare Figures 3A,C with Figure 3B). Despite the fact that more inhibition is typically associated to less activity, it is well-established that resonant neurons, as Hodgkin Huxley model, can exhibit inhibition-induced spiking (Izhikevich, 2003). For neuronal populations and the neural mass model, the transition from DS to AS occurs roughly when the receiver pulses faster than the sender, whether it is due to the internal (Figures 4B,C) or the external factor (Figures 4E,F). Nevertheless, this effect cannot be observed for the 3-neuron motif (Figures 4A,D), when the system is uncoupled; the Receiver always pulses at the same frequency or faster than the Sender (Figures 3A–D). This is due to the fact that, unlike other motifs, the Sender neuron is not subjected to any kind of inhibition. This means that in the uncoupled configuration, in the absence of inhibition at the receiver and same external current both neurons are identical and consequently ω_*R*_ = ω_*S*_.

## 4. Discussion

In this paper we have studied the effects that a change in the inhibitory conductance or an increase in the external forcing play in the transition from delayed to anticipated synchronization in neuronal circuits. Our study covers three cases of unidirectionally coupled systems: two Hodgkin-Huxley (HH) neurons where the receiver neuron is coupled to an inhibitory interneuron, two populations and two neural mass (NM) models. The results obtained for the HH neurons and the neuron populations confirmed that, when changing the inhibitory conductance in the receiver side, the systems can undergo a delayed to anticipated synchronization transition (Matias et al., 2011, 2014). Similar results were obtained when analyzing two coupled neural mass (NM) models. Interestingly, we found a second mechanism that yields similar results. If we fix the inhibitory conductance in all cases but change the external input (external current for the HH and NM models or the Poisson rate in the populations) the system can also undergo a DS to AS transition.

To unveil if the two mechanisms are independent or not, we studied how the pulsating frequency of an isolated system (a pair of excitatory-inhibitory neurons, a single population containing excitatory and inhibitory neurons or a NM model) changes when changing the inhibitory conductance or the external forcing. We found for the excitatory-inhibitory pair and the NM model that an increase in the inhibitory conductance indeed increases the pulsating frequency (see Figures 3A–C), as happens when we increase the external current. However, for the parameters we have used for the populations, an opposite behavior is observed: an increase in the inhibitory conductance decreases the oscillating frequency of the population. These results suggest that the two mechanisms might be independent although a more exhaustive analysis is necessary. Nevertheless, we propose that the unifying mechanism that promotes the transition between DS and AS in the sender-receiver motif is indeed an increase of the internal dynamics of the receiver system. This was observed at the three scales that we examined. This result is in agreement with previous studies of AS in a theoretical framework with the explicit time-delay in Equation (1) (Hayashi et al., 2016) and in simplified neural models without the explicit time-delay (Pyragienè and Pyragas, 2013, 2015; Dima et al., 2018).

Previous studies have shown that a biologically plausible mechanism for anticipation of pre-synaptic inputs is a combination of short-term synaptic depression (STD) and intrinsic spike-frequency adaptation (SFA) (Puccini et al., 2006, 2007). In the presence of both STD and SFA the post-synaptic system approximately computes the derivative a pre-synaptic stimuli which allows it to anticipate temporally incoming synaptic inputs. Their findings quantitatively agree with experimental results on anticipatory responses to moving stimuli in the primary visual cortex (Jancke et al., 2004; Puccini et al., 2007). We propose that the DS-AS transition studied here could be mediated by any parameter that turns the internal dynamics of the free-receiver faster and could also account for delay compensation in cortical systems. Specially the DS-AS transition could explain commonly reported short latency in visual systems (Orban et al., 1985; Nowak et al., 1995; Kerzel and Gegenfurtner, 2003; Jancke et al., 2004; Puccini et al., 2007; Stepp and Turvey, 2010, 2017; Martinez et al., 2014). Therefore, our results open new possibilities to further experimental investigation of anticipatory dynamics in neuronal systems.

## Data Availability

The raw data supporting the conclusions of this manuscript will be made available by the authors, without undue reservation, to any qualified researcher.

## Author Contributions

LD, FM, PC, MC, and CM designed the research and wrote the article. LD, AdS, and AA performed the simulations. LD performed the data analysis.

### Conflict of Interest Statement

The authors declare that the research was conducted in the absence of any commercial or financial relationships that could be construed as a potential conflict of interest.
